# Malignant gastrointestinal neuroectodermal tumor: A case report and review of the literature

**DOI:** 10.3892/ol.2014.2524

**Published:** 2014-09-11

**Authors:** JIE KONG, NAN LI, SHIWU WU, XINGMEI GUO, CONGYOU GU, ZHENZHONG FENG

**Affiliations:** Department of Pathology, The First Affiliated Hospital of Bengbu Medical College, Bengbu Medical College, Bengbu, Anhui 233000, P.R. China

**Keywords:** malignant gastrointestinal neuroectodermal tumor, stomach, S-100 protein, HMB45, *EWSR1*

## Abstract

Malignant gastrointestinal neuroectodermal tumor (GNET) is a rare soft tissue sarcoma, previously referred to as clear cell sarcoma-like gastrointestinal tumor (CCSLGT) and also commonly reported in the literature as clear cell sarcoma of the gastrointestinal tract (CCS-GI). The current study reports a case of GNET arising in the stomach of a 17-year-old male, who presented with symptoms of fatigue, anemia and low temperature. Examination with positron emission tomography-computed tomography revealed a soft tissue mass in the gastric antrum. Subsequently, radical distal gastric resection was performed, and the mass measured 6.0×4.0×3.5 cm^3^. Histopathological analysis revealed that the tumor cells were arranged in nests and focally formed fascicular, pseudopapillary, pseudoalveolar and rosette-like growth patterns. Osteoclast-like giant cells were also observed. Immunohistochemically, the tumor cells were positive for S-100 protein, vimentin and BCL-2, and negative for HMB45, Melan-A, CD117, CD34 and CD99. Additionally, the osteoclast-like giant cells were positive for CD68. Fluorescence *in situ* hybridization demonstrated *EWSR1* gene rearrangement. After 10 months of follow-up, no evidence of recurrence or metastasis was observed. As GNET is currently classified differently and under various names in the literature, the information provided by this case study and review is predicted to be useful towards the accurate diagnosis, treatment and prognosis of this rare tumor type.

## Introduction

Malignant gastrointestinal neuroectodermal tumor (GNET), named by Stockman *et al* (1) in 2012, is a rare tumor of the gastrointestinal tract. It has been previously referred to as clear cell sarcoma-like gastrointestinal tumor (CCSLGT) (1,2) or clear cell sarcoma-like tumor with osteoclast-like giant cells of the gastrointestinal tract (3–5), and is also commonly reported in the literature as clear cell sarcoma of the gastrointestinal tract (CCS-GI) (6–15). Clear cell sarcoma (CCS) was initially described by Enzinger (16) in 1965, and often occurs in the distal limb deep soft tissue, particularly in tendons and aponeuroses; therefore, it is also known as clear cell sarcoma of the tendons and aponeuroses (16,17). Subsequently, researchers have demonstrated that the tumor has obvious characteristics of melanocytic differentiation, but differs from malignant melanoma with respect to clinical, genetic and biological factors. Therefore, in 1983, CSS was renamed as malignant melanoma of soft parts by Chung and Enzinger (18).

In 1993, Ekfors *et al* (17) reported a case of CCS in the duodenum, which was the first visceral case reported. Following this, in 1998, Kothaj *et al* (19) reported the initial case of CCS of the stomach. Subsequently, a number of CCS cases in the gastrointestinal tract were reported successively, the majority of which lacked melanocytic differentiation features, and were commonly reported as CCSLGT. Stockman *et al* (1) retrospectively analyzed 16 cases of CCSLGT and observed that the tumor exhibited neural differentiation potential; therefore, the authors suggested GNET as a more appropriate name for this tumor type, an assessment that we agree with. The current study reports a case of GNET in the stomach and reviews the literature, focusing on similar cases and tumor classification. Written informed consent was obtained from the patient’s family.

## Case report

### Clinical features

A 17-year-old male was admitted to the Department of Gastrointestinal Surgery at the First Affiliated Hospital of Bengbu Medical College (Bengbu, China) with a two-month history of fatigue, discontinuous low temperature and anemia. The patient initially felt weakness in the limbs, which was particularly apparent following physical activity and, subsequently, weakness and fatigue affected the whole body. Concomitantly, the patient exhibited mild symptoms of abdominal distension and melena. On examination, a hemoglobin level of 66 g/l was recorded (normal reference range, 110–160 g/l), therefore, blood transfusion therapy was administered; however, no clear response was observed. Subsequently, positron emission tomography-computed tomography examination revealed a soft tissue mass in the gastric antrum, which exhibited increased fluoride deoxidization glucose. The gastroscopy results revealed irregular hyperplasia at the gastric antrum, as well as ulcers and signs of necrosis. Due to these observations, a radical distal gastric resection was performed. During the surgery, several swollen lymph nodes were identified and dissected; these were located under the pylorus and around the common hepatic artery, left gastric artery and celiac artery.

### Gross and histological features

**Figure 1:**
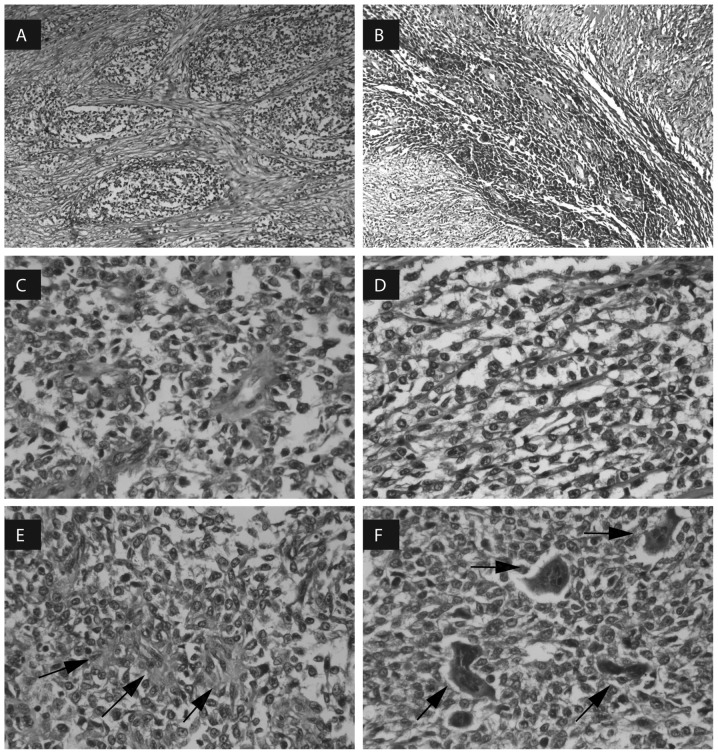
Morphological characteristics of the tumor cells following hematoxylin-eosin staining. (A) The tumor cells were arranged in nests surrounded by fibrous connective tissue (magnification, ×100) and (B) the spindle tumor cells were arranged in a fascicular pattern (magnification, ×100). Tumor cells focally formed (C) a pseudoalveolar architecture (magnification, ×400) and (D) a pseudopapillary architecture (magnification, ×400). Arrows indicate (E) rosette-like growth patterns (magnification, ×400) and (F) multinucleated osteoclast-like giant cells (magnification, ×400).

Pathological examination of the resected stomach specimen revealed a gray ulcerated mass, measuring 6.0×4.0×3.5 cm^3^. The microscopic examination demonstrated that the tumor had invaded the serosa layer of the stomach. No tumor tissue was apparent in the swollen lymph nodes. The medium-sized and round, oval or spindle-shaped tumor cells were arranged in a nest, and focally formed fasciculate, pseudopapillary, pseudoalveolar and rosette-like growth patterns (Fig. 1A–E) surrounded by fibrous connective tissue. A number of multinucleated osteoclast-like giant cells were also identified (Fig. 1F), composed of five to 20 nuclei, which was the most prominent morphological characteristic. The tumor cells consisted of weak eosinophilic cytoplasm, vacuolated nuclear chromatin and basophilic nucleoli.

### Immunohistochemical and molecular genetic features

**Figure 2:**
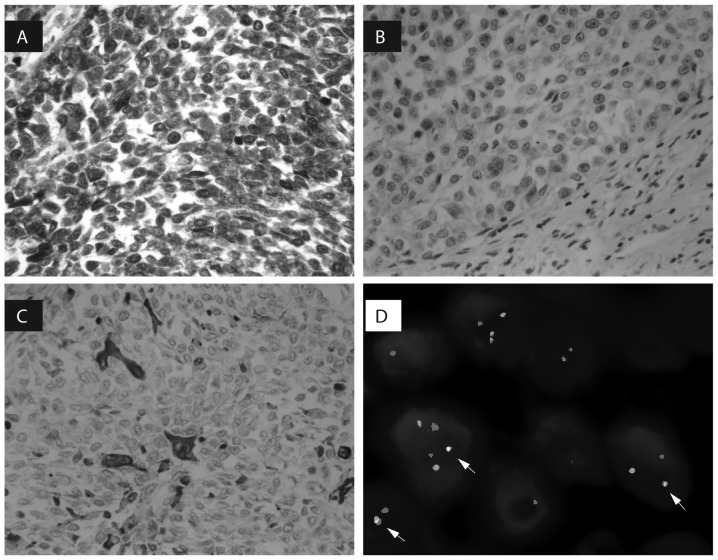
Immunohistochemical and fluorescence *in situ* hybridization results. (A) S-100 protein was diffusely expressed in tumor cells (magnification, ×400). (B) Tumor cells were negative for HMB45 (magnification, ×400) and (C) the osteoclast-like giant cells were positive for CD68 (magnification, ×400). (D) Fluorescence *in situ* hybridization results revealed genetic disruption of *EWSR1* (arrows indicate the abnormal signals).

The tumor cells were diffusely positive for S-100 protein (Fig. 2A), strongly positive for vimentin, and focally positive for BCL-2 and CD57. By contrast, the tumor cells were negative for HMB45 and Melan-A, which are markers of melanocytic differentiation (Fig. 2B). The osteoclast-like giant cells were positive for CD68 (Fig. 2C). In addition, several other indicators were negative, including cytokeratin, smooth muscle actin, desmin, CD117, CD34, MyoD1, CD99, calponin, WT-1, CD21, CD23, CD35, D2-40, CD1α, EMA, synaptophysin, CD56, neuron-specific enolase, CD30 and ALK1. Furthermore, 5–10% of tumor cells exhibited Ki-67 expression. At the genetic level, fluorescence *in situ* hybridization demonstrated *EWSR1* gene rearrangement. The proportion of cells exhibiting an abnormal signal indicating the genetic disruption of *EWSR1* was 71% (Fig. 2D).

## Discussion

In a review of the literature, the majority of CCS-GI cases were found to be cases of GNET; however, we hypothesize that GNET and CCS represent two distinct tumor types. Where CCS tumors are arranged in nests or fascicles (16) and have multinucleated Touton-like giant cells (2), GNETs exhibit alternative arrangements in addition to nests and fascicles, including a number of multinucleated osteoclast-like giant cells. Immunohistochemically, CCS tumors have been demonstrated to express melanocytic differentiation-related markers, including HMB45, Melan-A and MiTF (2,20,21), which are not a characteristic of GNET. CCS tumors have been associated with a balanced chromosome translocation t(12;22)(q13;q12), which results in the fusion of *EWSR1* (located at 22q12) and *ATF1* (located at 12q13) (2). GNETs also exhibit *EWSR1* gene rearrangements, confirming that this is not a tumor-specific characteristic. Furthermore, such rearrangements have been detected in Ewing’s sarcoma (22), angiomatoid fibrous histiocytoma (23), primary pulmonary myxoid sarcoma (23) and hyalinizing clear cell carcinoma of the salivary gland (23).

A search of the relevant literature revealed a total of 39 published case reports that may be considered as GNET, occurring in the stomach (eight cases) (1,4,5,7,10), ileum (14 cases) (1,4,11–13,15,22), jejunum (nine cases) (1,3,4,6,8,14), colon (three cases) (1,22) and small intestine (five cases) (1,12). Overall, GNETs (including the present case) span a wide age range of 10–81 years (median, 36 years; mean, 39 years) and the male to female ratio is 18:22. Patients commonly exhibit symptoms of abdominal pain and abdominal distension or weight loss, and a few patients present with anemia, melena, fever or other symptoms. The mean tumor size is 5.49 cm (range, 2.4–15.0 cm). Histologically, GNET consists of epithelioid or oval-to-spindle tumor cells arranged in sheets or nests, which focally form pseudoalveolar, pseudopapillary, microcystic, fascicular, cord-like or rosette-like growth patterns. A number of multinucleated osteoclast-like giant cells have also been observed. GNETs express a primitive neural phenotype, such as positivity for S-100 protein, SOX10, NSE, synaptophysin, CD56 and NB84, with no expression of melanocytic markers. Notably, BCL-2 was positively expressed in the present case. Under the electron microscope, GNET exhibits evidence of neural differentiation, including multiple interdigitating cell processes containing dense core granules and clear vesicles resembling synaptic bulbs (1). At the molecular genetic level, GNET is associated with *EWSR1* gene rearrangements, which results in the fusion of *EWSR1* and *ATF1*, or *EWSR1* and *CREB1* (1,22,23). The characteristics of the case reported in the current study are consistent with those of other GNET cases.

The various diagnoses of GNET include gastrointestinal stromal tumor (GIST), alveolar rhabdomyosarcoma, synovial sarcoma and malignant peripheral nerve sheath tumor (MPNST). However, characteristic properties of each diagnosis have been observed. GIST is positive for CD34 and CD117, alveolar rhabdomyosarcoma is positive for desmin and MyoD1, and is associated with a t(2;13)(q35;q14) chromosome translocation and synovial sarcoma often expresses the epithelial membrane antigens, CK7, CK19, and CD99. Furthermore, almost all synovial sarcomas exhibit the constant translocation t(X;18)(p11;q11). MPNST is positive for the S-100 protein, Leu-7, PGP9.5 and myelin basic protein. The *NF1* gene inactivation may be used to confirm the diagnosis of MPNST. The most common treatment for patients with GNET is excision of the tumor. Following the initial surgical resection, five of 40 cases exhibited liver metastasis (3,4,8,11,22), whereas 19 of 40 cases showed lymph node metastasis at the time of diagnosis (1,4, 5,12–14,22). In total, eight of 40 cases succumbed to the disease (1,4). After 10 months of follow-up, no evidence of recurrence or metastasis was identified in the present case.

In general, the diagnosis of GNET is based on the histological, immunohistochemical and molecular genetic features. The information presented in this study contributes further much required knowledge of GNET, which may aid in the diagnosis, treatment and prognosis of the tumor; however, clinical data from additional patients are required due to the rarity of the tumor.

## Abbreviations

GNET: malignant gastrointestinal neuroectodermal tumor
CCSL-GI: clear cell sarcoma-like gastrointestinal tumor
CCS-GI: clear cell sarcoma of the gastrointestinal tract
CCS: clear cell sarcoma
GIST: gastrointestinal stromal tumor
MPNST: malignant peripheral nerve sheath tumor
